# Genetic diversity of oral streptococci in the guinea pig as assessed by sequence analysis of the 16S rRNA and *groEL* genes

**DOI:** 10.1007/s12223-021-00936-3

**Published:** 2021-12-21

**Authors:** Jarosław Król, Aneta Nowakiewicz, Alicja Błaszków, Maria Brodala, Adrianna Domagała, Anna-Nicole Prassol, Dominika Sławska, Julita Wojtynia

**Affiliations:** 1 Department of Pathology, Faculty of Veterinary Medicine, Wroclaw University of Environmental and Life Sciences, St. Norwida 31, 50-375 Wrocław, Poland; 2 Sub-Department of Veterinary Microbiology, Faculty of Veterinary Medicine, University of Life Sciences in Lublin, St. Akademicka 12, 20-033 Lublin, Poland; 3 Student of the Faculty of Veterinary Medicine, University of Environmental and Life Sciences, Wrocław, Poland

## Abstract

The aim of the present study was to characterize bacteria of the genus *Streptococcus* isolated from the oral cavity of the guinea pig as well as to assess the significance of these microorganisms as potential veterinary and human pathogens. Sixty-two streptococcal isolates recovered from 27 clinically healthy guinea pigs were examined genotypically by sequencing the 16S rRNA and *groEL* genes. Among these isolates, only 13 could be assigned to a species described previously (mainly *Streptococcus parasanguinis*, *S*. *mitis* and *S. suis*), and the majority of the remaining ones differed considerably from the streptococcal species known to date (16S rRNA and *groEL* sequence similarities were < 97% and < 87%, respectively). Based on 16S rRNA sequences, these unidentified isolates were divided into seven groups (clades), of which clades I through III comprised most of the isolates examined and had also the widest distribution among guinea pig colonies. Upon *groEL* gene sequence analysis, however, members of the three clades grouped together without forming such distinct clusters. The remaining clades distinguished by 16S rRNA sequencing could also be discerned by the second gene, and they contained only a few isolates often restricted to one or a few animal colonies. The present work reveals that the guinea pig mouth is inhabited by a vast number of phylogenetically diverse, so far unrecognized populations of streptococci, most of them being apparently host-specific genomospecies. On the contrary, *S*. *parasanguinis* and *S*. *mitis* are also common human commensals and *S*. *suis* is a well-recognized zoonotic pathogen.

## Introduction

For years, guinea pigs (*Cavia porcellus*) have been among the most frequently used experimental animals. They have often been involved in studies of allergy, hearing problems, and respiratory and nutritional diseases (Shomer et al. 2015). In addition, the animals are now becoming very popular pets kept in households throughout the world. According to data collected by the European Pet Food Industry Federation, an estimated 28.56 million of small mammals (rabbits and rodents, including guinea pigs) are owned as pets in Europe (FEDIAF 2019). Guinea pigs are also not infrequent patients of veterinary clinics (Meredith 2015), and dental diseases (tooth elongation, tooth fractures, periodontitis, and odontogenic abscesses) belong to the most frequently diagnosed health disorders in these animals (Minarikova et al. 2015). Because such problems may often be complicated by various oral microorganisms, the treatment of these disorders usually requires a combination of surgical and pharmacological therapy (Minarikova et al. 2016). Bacteria inhabiting the oral cavity of the guinea pig can also cause, though sporadically, bite wound infections in humans (Abrahamian and Goldstein 2011; Lion et al. 1995). For these reasons, study on the composition of guinea pig oral microorganisms may be of interest from clinical, ecological, and epidemiological viewpoints.

The genus *Streptococcus* comprises a large number of Gram-positive cocci occurring predominantly as commensals on the mucosal membranes of humans and various animals. Currently, the genus includes more than 130 recognized species (http://www.bacterio.net/streptococcus.html), varying greatly in the host range and pathogenic properties. Some species (e.g. *S*. *pneumoniae*, *S*. *agalactiae*, *S*. *suis*) can inhabit and infect multiple hosts, whereas many others are restricted to humans or a single animal host. In small mammals (rodents and lagomorphs), some reportedly host-specific *Streptococcus* species include *S*. *cuniculi* (in wild rabbits), *S*. *ratti*, *S*. *ferus*, *S*. *orisratti* (in rats), and *S*. *criceti* (in hamsters) (Coykendall 1977; Vela et al. 2014; Zhu et al. 2000). Quite recently, a group of streptococci from fecal samples of guinea pigs was studied in the Netherlands. One of these isolates (designated as Cavy grass 6), capable of degrading cellobiose, was described to represent a novel species, *S*. *caviae* (Palakawong Na Ayudthaya et al. 2017). This microorganism is thought to contribute to the production of organic acids from plant-based food in the lower gastrointestinal tract. However, little is known about the composition of the streptococcal microbiota in the upper digestive tract of this animal. Therefore, the aim of the present study was to perform, by means of 16S rRNA and *groEL* gene sequencing, a phylogenetic analysis of streptococci isolated from the oral cavity of guinea pigs. In addition, the pathogenic potential of these bacteria for their host and for humans was assessed.

## Material and methods

### Isolation and preliminary identification of bacteria

Swabs were collected from the oral cavity of 27 adult, clinically healthy guinea pigs kept as pets by private owners in 8 separate and mutually unrelated colonies (designated A through H). This material was inoculated on Tryptone Soya Agar (Oxoid Ltd., Basingstoke, UK), supplemented with 5% defibrinated sheep blood. Plates were incubated aerobically for 24 h at 37 °C and, after transferring of putative streptococcal colonies onto fresh blood agar plates, re-incubated for the next 48 h. Obtained microorganisms were tentatively identified by colony and cell morphology, Gram stain, hemolytic properties, and catalase activity. Up to five isolates, varying in growth characteristics (diameter and colour shade of colonies, size of hemolysis zone), were collected from one animal.

### Assessment of biochemical properties

For a selected group of isolates representing all main genogroups (clades) recognized in the present study, biochemical tests using the API 20 STREP identification system (bioMérieux, Marcy l’Etoile, France) were performed.

### DNA extraction and genotypic studies

The isolation of bacterial DNA was carried out using the procedure described in a previous work (Król et al. 2016), except not adding lysostaphin to the extraction buffer. Molecular identification and phylogenetic studies of streptococcal isolates were based on nucleotide sequence analysis of the 16S rRNA and *groEL* genes. Amplification and sequencing of the 16S rRNA were accomplished using universal bacterial primers, 16S-27f (5′-AGAGTTTGATCMTGGCTNAG-3′) and 16S-907r (5′-CCGTCAATTCMTTTRAGTTT-3′) (Harmsen et al. 2003), giving a product of approximately 927 bp. For amplification and sequencing of the *groEL* gene, the primers streptogroELd and streptogroELr were used (Glazunova et al. 2009), amplifying a PCR product of ~ 770 bp. The PCRs involved 40 cycles of denaturation (94 °C for 30 s), annealing (52 °C for 30 s), and elongation (72 °C for 1 min). PCR products were purified and sequenced (on both DNA strands) using the DYEnamic ET terminator cycle sequencing kit ABI Prism™ (Amersham Biosciences Europe GmbH, Germany). For species identification, sequence similarities ≥ 99% (for the 16S rRNA gene) or ≥ 97% (for the *groEL* gene) with known sequences deposited in GenBank (as compared by the BLAST algorithm) were considered as conclusive. Phylogenetic analyses were performed using the MEGA version 3.1 software (Kumar et al. 2004). Dendrograms were generated by the Unweighted Pair Group Method with Arithmetic Mean (UPGMA).

### GenBank accession numbers for new sequences determined in the present study

Twenty-six representative sequences obtained within this study (13 sequences each of the 16S rRNA and *groEL* genes) were deposited in GenBank under the following accession numbers: MW045804 through MW045816 (16S rRNA) and MW045167 through MW045179 (*groEL*).

## Results

### Isolation and identification of bacteria

Bacterial culture revealed the presence of supposed streptococcal colonies in all the 27 guinea pigs tested, and the number of such colonies varied greatly among individuals (from few to abundant). The colonies were very small, ranging from 0.3 to 0.8 mm in diameter (after 24 h of incubation), translucent, and surrounded with a zone of alpha hemolysis. No catalase activity was found. Microscopic examination revealed Gram-positive cocci arranged in short chains.

Based on sequence analysis of the 16S rRNA gene, a total of 62 isolates were assigned to the genus *Streptococcus*. However, only 13 isolates achieved the stipulated 99% level of similarity of that gene to known sequences, being identified as *Streptococcus parasanguinis* (5 isolates), *S*. *mitis* (3), *S*. *suis* (2), *S*. *caballi* (1), *S*. *cristatus* (1), and *S*. *henryi* (1). For four *S*. *parasanguinis* and all *S*. *mitis* and *S*. *suis* isolates, this identification was confirmed by sequence analysis of the *groEL* gene (sequence similarity ≥ 97%). In addition, one isolate (B-S4/1) was recognized as belonging to the *Streptococcus ovis*/*minor* group (98.2% similarity of the 16S rRNA gene). The remaining 48 isolates (77,4%) displayed a much lower level of sequence homology of the 16S rRNA and *groEL* genes (< 97% and < 87%, respectively) and, consequently, could not be assigned to any of the known streptococcal species.

### Sequence analysis of the 16S rRNA gene

**Fig. 1:**
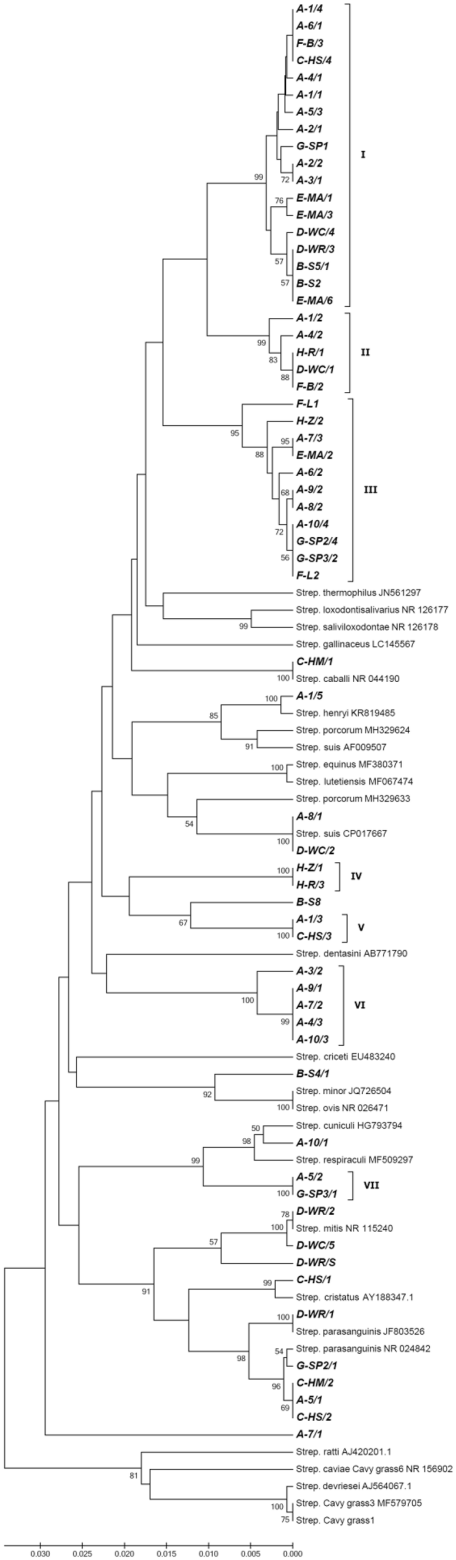
Phylogenetic tree of 89 *Streptococcus* strains (including 62 isolates determined in the present study) and 27 selected streptococcal strains (sequences retrieved from GenBank and provided with the corresponding accession numbers), based on 16S rRNA gene sequences (~ 900 bp fragments). The tree was constructed using the UPGMA method. The sequences indicated in bold correspond to isolates obtained in the present work. The designations A through H in the name of an isolate denote particular colonies of guinea pigs examined

A phylogenetic analysis of 16S rRNA sequences was carried out on 89 sequences, including 62 isolates obtained in the present study and 27 sequences retrieved from GenBank (corresponding to 22 defined species and 2 unidentified streptococcal isolates). A phylogenetic tree constructed by the UPGMA method is presented on Fig. 1. Sequences of the 48 unidentified isolates determined in this study were divided into 7 clades (designated I through VII). In addition, three isolates (A-10/1, A-7/1, and B-S8), presenting a lower level of similarity to any of these clusters, were placed separately in the dendrogram. Clade I comprised 18 isolates originating from animals living in all but one colony (A–G). Members of this group showed a 99.1–100% sequence similarity among each other (up to 8 mismatches on a total of 880 nucleotides compared) and only 96.1–97.0% similarity with their best-matching taxon in GenBank (i.e. *Streptococcus saliviloxodontae* NR_126178, QC 99%, *E*-value = 0.0). Clade II consisted of five isolates recovered from animals belonging to four colonies. For this group, *S*. *saliviloxodontae* was also the best hit in the BLAST search (with a similarity of 96.9–97.2%), but they differed by 19–22 nucleotides (97.5–97.8% sequence similarity) from most members of the clade I. Another group of streptococci analysed in the present work, containing 11 isolates originating from 5 animal colonies, was classified within clade III. These isolates appeared to be more distantly related to those of clade I, displaying only a 96.1–96.7% sequence similarity (29–34 mismatches out of 880 nucleotides) with them. Using the BLAST search, various species have been indicated as the best-matching taxa, i.e. *S*. *saliviloxodontae* (96.0–96.5% sequence similarity), *S*. *equinus* (95.0–95.3%), and *S*. *suis* (94.9–96.1%).

The remaining clusters, designated on the basis of the 16S rRNA gene, showed a low level of relatedness with members of the three above-described groups and were represented by a few isolates only. Clades IV, V, and VII comprised two isolates each; the best-matching taxa, obtained by the BLAST search, were *S*. *saliviloxodontae* (with a sequence similarity of 95.0%), *S*. *gallinaceus* (95.8%), and *S*. *cuniculi* (96.9–97.5%), respectively. The clade VI consisted of 5 streptococcal isolates originated from guinea pigs living in one colony; based on nucleotide sequences of the 16S rRNA gene, members of this cluster showed the highest similarity to *S*. *dentasini* (95.2–95.8%).

### Sequence analysis of the groEL gene

**Fig. 2:**
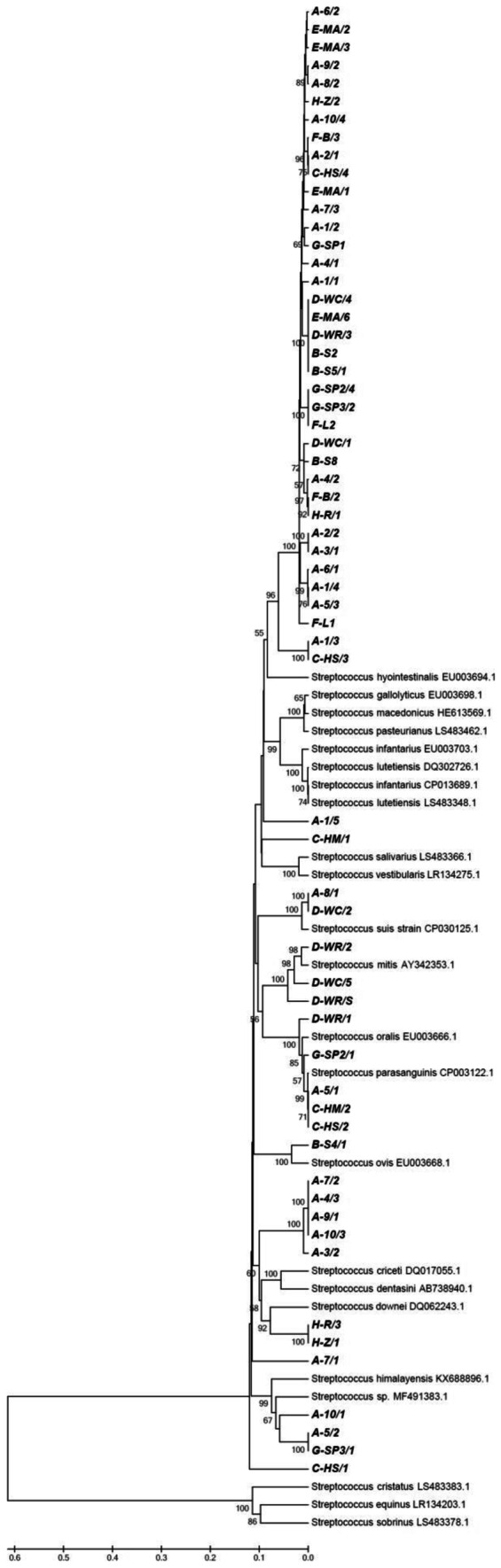
Phylogenetic tree of 85 *Streptococcus* strains (including 62 isolates determined in the present study) and 23 selected streptococcal strains (sequences retrieved from GenBank and provided with the corresponding accession numbers), based on *groEL* gene sequences (~ 740 bp fragments). The tree was constructed using the UPGMA method. The sequences indicated in bold correspond to isolates obtained in the present work. The designations A through H in the name of an isolate denote particular colonies of guinea pigs examined

Analysis of *groEL* gene sequences largely confirmed the genetic diversity and distinctness of the streptococcal population inhabiting the mucosal membrane of guinea pig’ mouth. The basic topology of the phylogenetic tree created on the basis of that gene was quite similar to that of the 16S rRNA gene (Fig. 2). However, the isolates belonging to clades I through III (based on 16S rRNA sequencing), although displaying some differences in nucleotide sequences of the *groEL* gene (they differed mutually by 0–40 nucleotides on a total of 740 nucleotides compared), did not make clearly separated lineages. Consequently, the three clades mentioned previously were merged together to form a large cluster comprising 35 isolates (including the isolate B-S8 which was placed separately in the 16S rRNA evolutionary tree). Members of this group, depending on the strain, had the highest sequence similarity to *S*. *hyointestinalis* (84.1–85.9%), *S*. *equinus* (84.4–84.8%), or *S*. *pasteurianus* (83.2%).

The remaining clades (IV through VII, as identified by sequence analysis of the 16S rRNA gene) could also be distinguished by *groEL* gene and contained the same respective bacterial isolates. For members of these four smaller groups, the closest relatives indicated by the BLAST search were *S*. *downei* (85.5%), *S*. *gallolyticus*/*S*. *macedonicus* (85.6%), *S*. *dentasini* (82.7–82.9%), and *S*. *respiraculi* (86.6%), respectively.

### Results of biochemical tests

Biochemical tests (using API 20 STREP) were performed in 25 out of 48 unrecognized isolates (Table 1). In general, the microorganisms analysed displayed weak to moderate metabolic activity. The production of leucine arylamidase was the most frequently detected reaction (in all but one of the isolates tested). Some other tests (the Voges-Proskauer reaction, esculin hydrolysis, production of α-galactosidase, β-galactosidase, or β-glucuronidase) were observed much less commonly and occurred irregularly among members of particular groups. Only a few isolates examined were capable of producing acid from the carbohydrates included in the identification system (lactose and/or trehalose).

**Table 1:** Biochemical characteristics of 25 unidentified streptococcal isolates originating from the oral cavity of guinea pigs, determined using API STREP system

Clade^a^ (number of isolates analysed biochemically)	Numerical code obtained with the API 20 Strep system (number of isolates)	Positive reactions
**I** (8)	0 000 000 (1) 0 040 000 (4) 1 040 000 (1) 1 050 000 (1) 1 250 410 (1)	None LAP VP, LAP VP, β-GAL, LAP VP, α-GAL, β-GAL, LAP, LAC, TRE
**II** (1)	0 040 000 (1)	LAP
**III** (6)	0 040 000 (2) 0 050 000 (1) 0 060 400 (1) 1 050 000 (1) 4 060 000 (1)	LAP β-GAL, LAP LAP, PAL, LAC VP, β-GAL, LAP ESC, LAP, PAL
**IV** (2)	1 040 000 (2)	VP, LAP
**V** (2)	5 250 000 (1) 5 251 000 (1)	VP, ESC, α-GAL β-GAL, LAP VP, ESC, α-GAL β-GAL, LAP, ADH
**VI** (4)	0 040 010 (2) 4 040 010 (1) 4 060 010 (1)	LAP, TRE ESC, LAP, TRE ESC, PAL, LAP, TRE
**VII** (2)	0 440 000 (1) 1 650 000 (1)	β-GUR, LAP VP, α-GAL, β-GUR, β-GAL, LAP

*ADH* arginine dihydrolase, *ESC* esculin, *α-GAL* α-galactosidase, *β-GAL* β-galactosidase, *β-GUR* β-glucuronidase, *LAC* lactose, *LAP* leucine arylamidase, *PAL* alkaline phosphatase, *TRE* trehalose, *VP* the Voges-Proskauer reaction

^a^Based on sequence analysis of the 16S rRNA gene

## Discussion

Guinea pigs represent a specific group of monogastric herbivores, differing from other animals with regard to anatomy of the gastrointestinal tract and physiology of food digestion. This may also be reflected in distinctive microbial community associated with the animal species. However, detailed studies have been mainly focused on the intestinal microbiota in the guinea pig (Crowley et al. 2017), and little is known about the composition of oral microbial populations, including streptococci.

In the present work, we carried out a phylogenetic analysis of streptococci isolated from the oral cavity of 27 guinea pigs kept in 8 unrelated colonies. To accomplish this, partial sequencing of the 16S rRNA and *groEL* genes was performed. Determination of 16S rRNA sequence is usually the first step in identification of a bacterial strain, allowing its recognition at the genus level (Glazunova et al. 2009). In addition, the 16S rRNA gene turned out to be a valuable tool for determining phylogenetic relationships among streptococci (Kawamura et al. 1995). However, results obtained from the sequencing of the 16S rRNA gene alone may be inconclusive owing to the fact that two or more bacterial species share more than 99% similarity of that gene (Kawamura et al. 1995). The second gene analysed (*groEL*), encoding the 60-kDa heat-shock protein, has also proved to be a good tool for determining interspecies relationships among streptococci (Glazunova et al. 2009). It is reported to be less conserved and more discriminatory than the former, and therefore useful in the differentiation of closely related species. Moreover, it was shown that evolutionary trees constructed based on the *groEL* gene demonstrate marked similarities to those derived from 16S rRNA sequences (Teng et al. 2002).

Our research revealed that the guinea pig carries a large number of streptococci varying greatly in genotypic properties. Results of the BLAST search as well as phylogenetic trees created by the UPGMA method with partial sequences of 16S rRNA and *groEL* genes confirmed the placement of all the isolates studied within the genus *Streptococcus*. Interestingly, only a small portion of these bacteria could be classified within known species (e.g. *S*. *parasanguinis*, *S*. *mitis*, *S*. *suis*) and the majority of the isolates (~ 77%) belonged to previously unknown evolutionary groups (clades). Partial sequence analysis of the 16S rRNA gene allowed for the recognition of as many as seven such lineages. Most of the unidentified isolates (34 out of 48), originating from 24 individuals representing all the colonies studied, belonged to the clades designated I through III (18, 5, and 11 isolates, respectively). These phylogenetic groups were relatively closely related to each other, showing 16S rRNA gene similarities of ~ 97.5% (clade I vs II) or ~ 96.5% (I vs III). For all these three clades, sequences of the nearest relatives indicated by the BLAST algorithm (most frequently, *S*. *saliviloxodontae*) differed by 3–4%. This level is below the commonly accepted thresholds of 16S rRNA gene sequence similarity used for the species delineation. According to a classical taxonomic study, two different bacterial species show at most 97% 16S rRNA gene sequence similarity (which corresponds to 70% DNA-DNA homology acknowledged as a boundary for species definition) (Stackebrandt and Goebel 1994). Subsequent experiments, based on an extended number of microbial strains, recommended an even higher threshold of 16S rRNA gene sequence similarity of 98.7–99.0% (Stackebrandt and Ebers 2006) or, more precisely, 98.65% (Kim et al. 2014) for this purpose. Considering these criteria, the three clades (I–III) distinguished in our study on the basis of 16S rRNA gene could represent three new, closely related species belonging to the salivarius group of the genus *Streptococcus*. Their nearest relative seems to be *S*. *saliviloxodontae* which was described to inhabit the oral cavity of the African elephant (Saito et al. 2014) and apparently not found elsewhere. Interestingly, this 16S rRNA gene–based separation into three clades (and, possibly, species) was not confirmed in the phylogenetic tree inferred from the *groEL* gene. The sequences corresponding to those three clades clustered in one large, quite coherent group which was clearly distinct from the remaining streptococcal sequences analyzed. Thus, the question whether the group constitutes only one heterogeneous species (as deduced from the *groEL* gene) or three closely related ones (based on 16S rRNA gene) remains open. Nevertheless, a relatively low sequence similarity of the *groEL* gene to the nearest relatives (~ 84%) supports the distinctness and taxonomic independence of these isolates. Given a very broad distribution of members of this group among guinea pigs (they were detected in the great majority of individuals examined), it is very possible that these bacteria show a high level of adaptation to the guinea pig and are most typical oral streptococci in this animal species.

The remaining clades of our unidentified streptococci from guinea pigs (designated IV–VII on the basis of 16S rRNA gene) were also found in the evolutionary tree inferred from comparison of *groEL* gene sequences. For a particular cluster, the same streptococcal isolates were assigned in both phylogenetic diagrams and these clades were independent of each other, sharing also a low level of similarity with *Streptococcus* species known to date. When compared to their closest relatives, the bacteria belonging to clades IV through VII had sequence similarities of the 16S rRNA gene at 95.0–97.5% and those of the *groEL* gene at 82.7–86.6%. For this reason, it is likely that these groups represent another four new streptococcal species. However, owing to the fact that particular clades were restricted in their occurrence to individual guinea pig colonies and contained only a few isolates, it is difficult to estimate whether these bacteria are specific for these animals or merely accidental inhabitants.

Noteworthy, none of the 62 isolates obtained from the guinea pig mouth within the present study had genotypic or phenotypic properties corresponding to those of *Streptococcus caviae*. Based on the phylogenetic tree created by comparison of 16S rRNA gene sequences, *S*. *caviae* (isolated from guinea pig faeces) differs distinctly from the oral streptococci originating from that animal host. In addition, as demonstrated on a representative group of our isolates tested on the API 20 STREP system, their biochemical activities are much weaker than those of *S*. *caviae*. For example, the oral isolates tested negative for mannitol, raffinose, and sorbitol. *S*. *caviae* is reported to be positive with these tests (Palakawong Na Ayudthaya et al. 2017). These results indicate that some streptococci associated with the guinea pigs can be not only host-specific but probably also restricted to particular anatomical regions.

Many streptococcal species are potential pathogens of their animal hosts and, occasionally, can cause zoonotic infections in humans. In the guinea pig, the most important members of this genus are *S*. *equi* subsp. *zooepidemicus* and *S*. *pneumoniae* which can cause suppurative lymphadenitis, abscesses, pneumonia, and fibrinopurulent inflammatory conditions of the serous membranes in their host (Shomer et al. 2015). As shown in the literature, some other streptococci occurring in the oral cavity of guinea pigs can also be potentially pathogenic for the animal and have been recovered from dental diseases, e.g. odontogenic abscesses (Minarikova et al. 2016). The authors of the last report detected, among other microorganisms, such streptococcal species as *S*. *anginosus*, *S*. *bovis*, *S*. *constellatus*, and *S*. *ratti*, as well as some unidentified α-hemolytic and β-hemolytic isolates. Unfortunately, the paper mentioned lacks a detailed description of the identification methods used by the investigators, leaving open the question of accuracy of the results and precluding a reliable comparison among various studies.

In our research, the major streptococcal pathogens of the guinea pig, i.e. *S*. *equi* subsp. *zooepidemicus* and *S*. *pneumoniae*, were not detected. This could be attributed to the uneven distribution of these bacteria among the animal populations or their lower prevalence in the oral cavity than in the respiratory tract. In fact, some reports describe subclinical upper respiratory tract carrier states of *S*. *pneumoniae* (with the possible transmission to humans) in over 50% guinea pig colonies (Shomer et al. 2015). By contrast, the present study has revealed that the guinea pig may also be a reservoir of other streptococci that are potential human pathogens, that is, *S*. *suis*, *S*. *parasanguinis* and *S*. *mitis*. In particular, *S*. *suis* is a well-established zoonotic pathogen with an increasing number of confirmed human cases in recent years. The most common clinical manifestations of infections caused by this microorganism in humans are meningitis and septicaemia, and the supposed route of entry is damaged skin (Gottschalk et al. 2007). *Streptococcus parasanguinis* and *S*. *mitis* can occasionally cause human infections (e.g. endocarditis), mainly in immunocompromised patients (Naveen Kumar et al. 2014).

In conclusion, the present study reveals that the guinea pig mouth is inhabited by a vast number of phylogenetically diverse populations of streptococci, representing several previously unrecognized genomospecies. In particular, a group of these microorganisms, distinguished by 16S rRNA and *groEL* genes and belonging to 1–3 closely related clusters, seems to be host-specific and occurs very frequently among guinea pig population. Further research using methods based on whole-genome sequencing (such as average nucleotide similarity — ANI) would be necessary to fully explain the evolutionary relatedness and taxonomic status of that group as well as other streptococci associated with the oral cavity of the guinea pig.

## Data Availability

Twenty six sequences obtained within this study (13 sequences each of the 16S rRNA and *groEL* genes) were deposited in GenBank under the following accession numbers: MW045804 through MW045816 (16S rRNA) and MW045167 through MW045179 (*groEL*).
